# Targeting of Lysosomal Pathway Genes for Parkinson's Disease Modification: Insights From Cellular and Animal Models

**DOI:** 10.3389/fneur.2021.681369

**Published:** 2021-06-14

**Authors:** Tetsuro Abe, Tomoki Kuwahara

**Affiliations:** Department of Neuropathology, Graduate School of Medicine, The University of Tokyo, Tokyo, Japan

**Keywords:** lysosome, α-synuclein, LRRK2, VPS35, ATP13A2, GBA

## Abstract

Previous genetic studies on hereditary Parkinson's disease (PD) have identified a set of pathogenic gene mutations that have strong impacts on the pathogenicity of PD. In addition, genome-wide association studies (GWAS) targeted to sporadic PD have nominated an increasing number of genetic variants that influence PD susceptibility. Although the clinical and pathological characteristics in hereditary PD are not identical to those in sporadic PD, α-synuclein, and LRRK2 are definitely associated with both types of PD, with *LRRK2* mutations being the most frequent cause of autosomal-dominant PD. On the other hand, a significant portion of risk genes identified from GWAS have been associated with lysosomal functions, pointing to a critical role of lysosomes in PD pathogenesis. Experimental studies have suggested that the maintenance or upregulation of lysosomal activity may protect against neuronal dysfunction or degeneration. Here we focus on the roles of representative PD gene products that are implicated in lysosomal pathway, namely LRRK2, VPS35, ATP13A2, and glucocerebrosidase, and provide an overview of their disease-associated functions as well as their cooperative actions in the pathogenesis of PD, based on the evidence from cellular and animal models. We also discuss future perspectives of targeting lysosomal activation as a possible strategy to treat neurodegeneration.

## Introduction

Parkinson's disease (PD) is the second most common neurodegenerative disease after Alzheimer's disease, affecting about 10 million people worldwide. PD is clinically characterized by bradykinesia, tremor, rigidity, and postural instability as well as olfactory abnormalities and sleep disturbances. The motor symptoms of PD are mainly attributable to the selective loss of dopaminergic (DA) neurons in the substantia nigra pars compacta (SNpc), causing dopamine deficiency (1). An important pathological hallmark in PD lesions is the intraneuronal inclusions called Lewy bodies that consist of aggregated α-synuclein phosphorylated at Ser129 residue (2–4). It is widely accepted that α-synuclein aggregates or oligomeric species spread to interconnected brain regions in a prion-like manner, although the processes are not fully understood (5).

Although the majority of PD cases (~90%) are sporadic, some forms of PD are hereditary and the responsible genes have been identified. *SNCA* encoding α-synuclein was the first gene identified, and the mutations in other genes such as leucine-rich repeat kinase 2 (*LRRK2*) and vacuolar protein sorting-associated protein 35 (*VPS35*) are also established as the cause for autosomal-dominant PD. On the other hand, genes associated with autosomal-recessive PD include *PRKN, PINK1*, and *ATP13A2* (6). Importantly, accumulating evidence has pointed to a greater contribution of genetic determinants in sporadic PD (7, 8). Especially, past meta-analyses of genome-wide association studies (GWAS) targeting sporadic PD have repeatedly identified two of the above familial PD genes—*LRRK2* and *SNCA*—as major risk factors, indicating that the impact of these two genes is more common in the general population (9–11). These GWAS for sporadic PD have succeeded in nominating a number of additional genes that were not identified from linkage analyses of familial PD cases, and *GBA1* in particular is the most representative of such genes.

Importantly, a significant proportion of PD-associated genes (*e.g., LRRK2, GBA1, ATP13A2, VPS35*, and *TMEM175*) have been functionally implicated in the endolysosomal system in cells (12–16). Especially, *GBA1* is well-known as a responsible gene for Gaucher disease, the most common lysosomal storage disorder. Moreover, the recent expansion of genetic, transcriptomic, and epigenetic studies in sporadic PD has nominated an increasing number of lysosomal pathway genes as a risk factor for PD (17–19). Endolysosomal dysfunctions are also frequently described in other neurodegenerative diseases such as Alzheimer's disease (AD), Huntington's disease (HD), frontotemporal dementia (FTD) and amyotrophic lateral sclerosis (ALS), all of which accompany neuronal accumulation of misfolded proteins (20, 21).

In addition to the evidence from genetics, the involvement of lysosomal dysfunction in PD has been implicated from pathological and biochemical studies using postmortem disease samples. The reduction in the immunoreactivity of lysosomal markers, such as LAMP1 and cathepsin D, was detected in PD and Lewy body disease (22, 23), and lysosomal breakdown, autophagosomal accumulation and the colocalization of autophagosomal markers with Lewy bodies were also detected in PD brains (24). Cathepsin D immunoreactivity has been shown to colocalize with α-synuclein pre-aggregates in nigral neurons in PD (25). The levels of lysosomal enzymes have been reported to be altered in cerebrospinal fluid and blood samples from PD patients (26–28). Thus, the role of lysosomes in PD pathogenesis is receiving increasing attention.

However, the detailed mechanisms on how lysosomal dysfunction leads to the neurodegeneration in PD remain largely elusive. There is a wide range of functions of PD-causative genes that are related to lysosomes, and much research has been focused on the elucidation of disease-related functions as well as the relationship among these genes. A common mechanism assumed by many researchers is that lysosomal dysfunction ultimately leads to α-synuclein accumulation and propagation in neurons. In fact, the role of lysosomes in α-synuclein degradation has long been attracted attention, and many studies on PD genes have also examined their effects on **α**-synuclein intracellular dynamics (i.e., metabolism, aggregation, secretion, and internalization).

In this article, we first summarize the current knowledge about the mechanisms of α-synuclein degradation in lysosomes, and then focus on the roles of other well-analyzed PD gene products, namely LRRK2, VPS35, ATP13A2 and GBA, in terms of their individual and co-operative regulations of endolysosomes and α-synuclein dynamics. Finally, we will discuss the potential of targeting endolysosomal system, especially the strategies to enhance lysosomal activity, in the future treatment of PD.

## α-Synuclein: The Central Effector Degraded in Lysosomes

Missense mutation in *SNCA* gene encoding α-synuclein was first identified in 1997 as a cause of autosomal-dominant PD (29). Later on, more mutations in *SNCA* gene have been identified to date, including A53T, A30P, E46K, H50Q, G51D, and A53E (29–34). Furthermore, gene triplication and duplication of *SNCA* locus without missense mutations have also been reported as a cause of familial PD (35–37). This means that the increase of α-synuclein level by itself is sufficient to develop PD, and therefore proper clearance of α-synuclein is required for the prevention of disease onset. Multiple lines of evidence have suggested that α-synuclein is degraded in two major proteolytic pathways: the ubiquitin-proteasome system (UPS) and the autophagy-lysosomal pathway (ALP) (38, 39). The metabolism in ALP has been the focus of much attention, especially in relation to the clearance of aggregated α-synuclein species.

Previous studies have shown that both extracellular and intracellular α-synuclein species are transported into lysosomes via the endosomal system or autophagy (40). It has been reported that α-synuclein is mainly degraded by cathepsins, especially cathepsin D, in lysosomes (41, 42). Cathepsin D level is shown to influence α-synuclein aggregation and toxicity *in vivo* (43). Treatment of cells with a lysosomal inhibitor bafilomycin A_1_ has been reported to not only affect α-synuclein metabolism but also to promote its propagation (44, 45).

Conversely, it has also been shown that the aggregated α-synuclein itself inhibits the function of lysosomes as well as other organelles. For example, α-synuclein pre-formed fibrils (PFFs) act on lysosomal membranes and cause its rupture (46–48). Another study has reported that α-synuclein impedes the lysosomal stress response mediated by the SNARE protein ykt6 (49). ykt6 is known as a regulator of ER-Golgi trafficking that is also reported to be disrupted by accumulated α-synuclein (50, 51), suggesting the possibility that the effect of α-synuclein on lysosomes is not necessarily direct. Collectively, it is assumed that lysosome inhibition exacerbates α-synuclein toxicity and α-synuclein accumulation in turn inhibits lysosomes, forming a vicious cycle that leads to the development of the disease.

Autophagy has also been established as a key mechanism regulating α-synuclein metabolism and toxicity. Macroautophagy is a major autophagy machinery that processes the degradation of a large portion of the cytoplasmic components through the formation of double-membrane structures called autophagosomes. The autophagosomes fuse with primary lysosomes to form autolysosomes where their contents are degraded and then either disposed or recycled back to the cell (52, 53). Inhibition of autophagosome-lysosome fusion by treatment with bafilomycin A_1_ or chloroquine enhanced α-synuclein release and transfer in human neuroglioma cells and rat primary cortical neurons (54, 55). In a mouse model of PD expressing human α-synuclein, impairment of macroautophagy under DA neuron-specifc knockout of *Atg7* gene caused the aggravation of neuropathology, although the behavior of mice was paradoxically improved (56). In humans, it has been reported that the majority of Lewy bodies (~80%) composed of α-syuclein in the SNpc of PD patients were strongly immonoreactive for LC3 (24), and similar observation for LC3 immunoreactivity was observed in Lewy bodies of dementia with Lewy bodies (DLB) patients (57). These reports collectively implicate the impaired macroautophagy in the pathogenetic processes involving α-synuclein, although we should note that there is little direct evidence of α-synuclein degradation by macroautophagy.

On the other hand, another type of autophagy called chaperone-mediated autophagy (CMA) has been considered as a possible mechanism of PD (58). CMA mediates the lysosomal degradation of a specific subset of soluble cytosolic proteins containing a KFERQ-like motif, which can be recognized by the cytosolic chaperone heat shock cognate protein 70 (Hsc70). Proteins targeted by Hsc70 are directly transported into the lysosomes for degradation through association with lysosome-associated membrane protein 2A (LAMP2A). It has been shown that wild-type α-synuclein can be degraded in CMA whereas mutant α-synuclein interferes with the lysosomal transport process in CMA, suggesting a possible link between defective CMA activity and PD (59).

**Figure 1:**
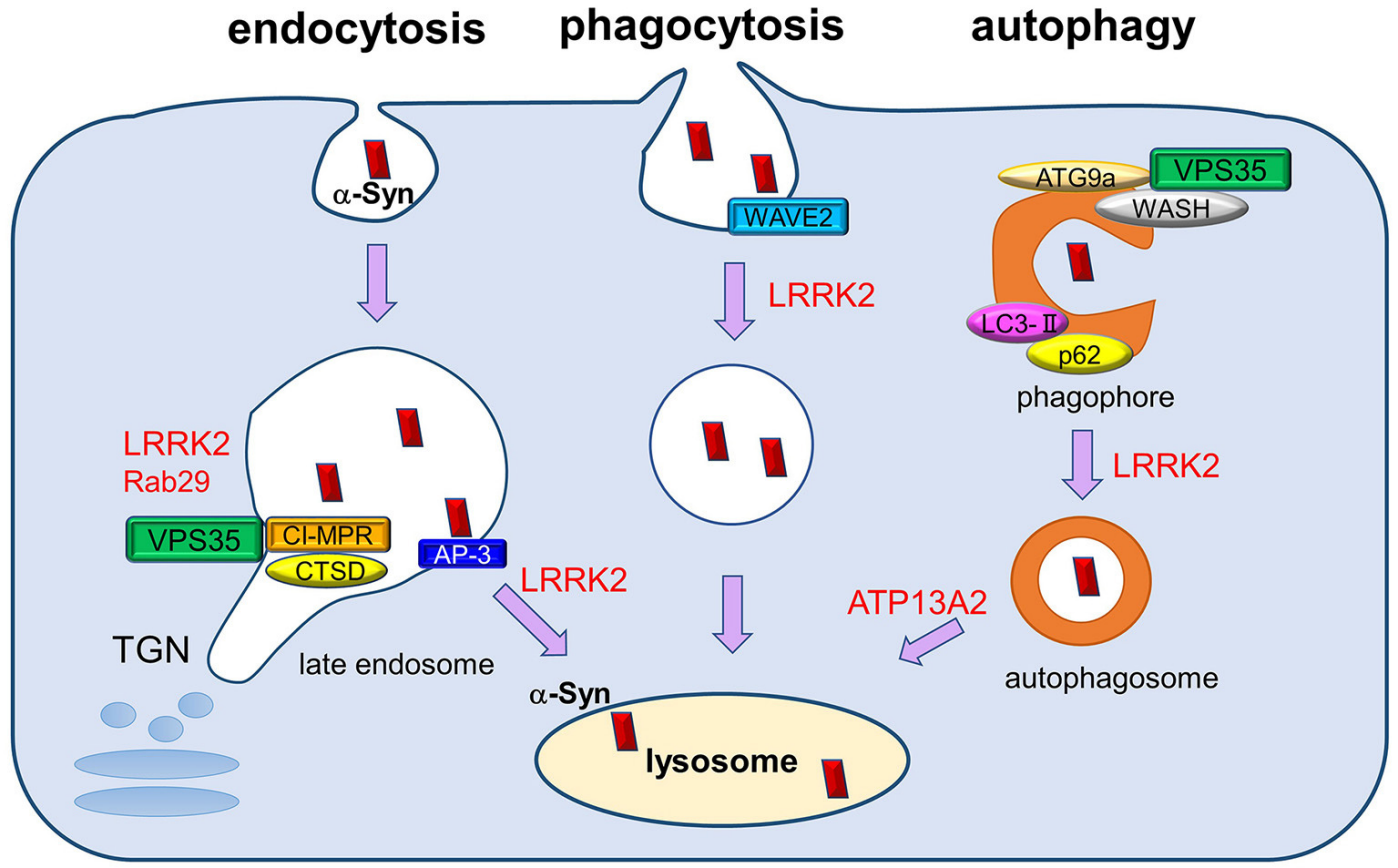
The roles of PD-associated proteins in endolysosomal pathways responsible for α-synuclein degradation. Extracellular and intracellular α-synuclein species (both soluble and aggregated) are transported into lysosomes for degradation through several pathways, including endocytosis, phagocytosis, and autophagy. PD-associated proteins VPS35, LRRK2, and Rab29 influence multiple steps of these degradation pathways, both individually and cooperatively. The retromer complex component VPS35 regulates the recycling of cathepsin D (CATD), the main lysosomal hydrolase responsible for α-synuclein degradation, by retrieving the lysosomal hydrolase receptor CI-MPR from endosome to the TGN. VPS35 pathogenic mutation may affect the recycling of CATD and thus impair α-synuclein degradation. LRRK2 and Rab29 further interact with VPS35 and regulate its function cooperatively. In endocytosis pathway, LRRK2 regulates AP-3-mediated intracellular recycling of lysosomal membrane proteins, whereas LRRK2 modulates the phagocytic activity by interacting with actin-cytoskeletal regulator WAVE2. VPS35 has been shown to function in macroautophagy pathway together with its interactors WASH complex and ATG9a, thereby regulating the transport of LC3-positive compartments. LRRK2 also regulates the autophagic flux, and ATP13A2 influences the clearance of autophagosomes. The perturbation of macroautophagy pathway is thought to contribute to the impaired degradation of α-synuclein, especially those of aggregated species.

Accumulating evidence has suggested that these ALP machineries may be modified by several PD-associated gene products, including LRRK2, VPS35, ATP13A2, and GBA. In the following sections, we will discuss the possible roles of these proteins in ALP and α-synuclein metabolism, focusing on the pathological relevance in PD (Figure 1).

## LRRK2: A Multifaceted Kinase in the Endolysosomal System

Mutations in *LRRK2* gene have been identified as the most common cause of autosomal-dominant PD (60, 61). LRRK2 is a large ~280 kDa protein and is widely expressed in human tissues including brains, although the expression is higher in the kidney, lung and immune cells (61–64). LRRK2 protein consists of multiple enzymatic and protein interaction domains including armadillo repeats (ARM), ankyrin repeats (ANK), leucine-rich repeats (LRR), Ras of complex (Roc), C-terminal of Roc (COR), kinase, and WD40 domains (61, 65, 66), suggesting that LRRK2 has diverse binding partners in distinct cellular pathways. LRRK2 has an ability to bind GTP through its ROC domain, and PD-associated mutations in LRRK2 have been shown to cause alterations in GTP binding and/or GTPase activity (64, 67, 68). A number of mutations in *LRRK2* gene have been reported so far (69), and the following mutations are well-validated: N1437H, R1441C/G/H, Y1699C, G2019S, and I2020T. These mutations are located either in the ROC domain (N1437H, R1441C/G/H), COR domain (Y1699C) or kinase domain (G2019S, I2020T). Among these, G2019S is the most prevalent, followed by R1441C/G/H (60, 61, 69–72). It has been shown that G2019S mutation in LRRK2 increases its intrinsic kinase activity (73), whereas ROC/COR domain mutants affect GTPase activity or GTP binding (64, 68). These findings implicate the important roles of both GTPase/GTP binding and kinase activities of LRRK2 in PD pathomechanisms. Recent structural analyses of LRRK2 on microtubules using cryo-electron tomography/microscopy have shown that the kinase and GTPase domains are in close proximity (74, 75), suggesting that the activities of both domains are not independent but influence each other.

Recent studies have accumulated evidence that LRRK2 phosphorylates a subset of Rab family GTPases, including Rab3, Rab8, Rab10, Rab29, and Rab35, in their switch-II regions (76–80). Rab GTPases are the major regulators of intracellular membrane trafficking (81). It has been shown that LRRK2 and its substrate Rab GTPases, especially Rab8 and Rab10, are readily recruited onto lysosomes that are stressed by lysosomal overload (82, 83) or by membrane damage (84, 85). Under lysosomal overload stress, LRRK2 and Rab8 act against lysosomal hypertrophy, whereas LRRK2 and Rab10 facilitate the release of lysosomal contents. Under lysosomal membrane-damaging stress, LRRK2 recruits the ESCRT-III component CHMP4B via Rab8a (85) or the motor adaptor protein JIP4 via Rab10/Rab35 (84) to damaged lysosomes for membrane repair. The lysosomal recruitment of LRRK2 is further regulated by Rab29 (also known as Rab7L1), another interactor and substrate of LRRK2 (82, 83). Studies in *C. elegans* neurons have suggested that the orthologs of LRRK2 and Rab29 both regulate axon termination, and double mutant analysis has revealed their functions in a same genetic pathway that involves the clathrin adaptor protein complex 3 (AP-3), an important regulator of Golgi-lysosome transport of lysosomal membrane proteins (86).

A variety of studies have also reported the relationship between LRRK2 and autophagy. Studies of *Lrrk2* KO mice have demonstrated the altered autophagic markers such as the autophagosome marker LC3-II and the autophagy substrate p62 (87, 88). The levels of these autophagic markers were changed in age-dependent and bi-phasic manners; LC3-II level was increased at 7 months of age but decreased at 20 months in *Lrrk2* KO mice, whereas p62 was decreased at 7 months and increased at 20 months (87). *In vitro* studies have shown that the knockdown of LRRK2 in neuroblastoma SH-SY5Y cells caused a marked increase in LC3-II and p62 levels (89). In contrast, another study has shown that the knockdown of endogenous LRRK2 in macrophage or microglial cells decreased LC3-II levels and autophagy flux (90). Thus, although these changes in the levels of autophagic markers indicate the important role of LRRK2 in the proper regulation of autophagic flux, the effects of LRRK2 on autophagy depend on the conditions such as cell type and experimental methodology, and the mechanism of how LRRK2 regulates autophagy still remains unclear.

As for the relationship between LRRK2 and CMA, it has been reported that LRRK2-G2019S inhibits CMA by affecting LAMP2A-mediated internalization of the substrate proteins like α-synuclein into lysosomes, which results in α-synuclein accumulation in neurons (91). Consistently, a significant reduction in CMA or lysosomal markers such as LAMP1, LAMP2A, Hsc70, and cathepsin D has been described in whole brains or SNpc of PD patients (22, 24, 92, 93). LRRK2 may additionally regulate the phagocytic activity in myeloid cells, where LRRK2 binds and phosphorylates the actin remodeling protein Wiskott-Aldrich syndrome protein family verprolin-homologous protein 2 (WAVE2), which is important for the efficient promotion of phagocytosis (94).

In neurons, LRRK2 physically and functionally interacts with the retromer complex component VPS35, which is also known as a causative gene product for hereditary PD. Retromer complex functions on endosomes to selectively transport cargo proteins to the *trans*-Golgi network (TGN) or plasma membranes (95), and intirectly regulates lysosomal functions, as described later. The LRRK2-VPS35 functional interaction in various experimental context was further modulated by a LRRK2-binding protein Rab29 (96). Another report has demonstrated that a pathogenic VPS35 mutation (D620N) influences LRRK2 kinase activity with unknown mechanism; that is, LRRK2 activity to phosphorylate its substrate Rab GTPases was significantly enhanced in VPS35[D620N] knock-in cells compared to those without VPS35 mutation (97). Collectively, there is considerable evidence that LRRK2 acts on endolysosomal system, although further analysis is needed to determine which of these functions is particularly important in PD pathogenesis.

## VPS35: An Indirect Regulator of Lysosomes

Mutations in vacuolar protein sorting-associated protein 35 (*VPS35*) gene are the genetic cause in *PARK17*, a locus for autosomal-dominant familial PD. Two independent groups have investigated Austrian and Swiss kindreds that develop PD and identified D620N mutation in VPS35 as the cause of the disease (98, 99). Patients with VPS35 D620N mutation have a mean age of onset in the 50s, and their clinical manifestations are similar to those of sporadic PD, such as resting tremor, bradycardia and L-DOPA reactivity (100, 101). Thus, although the presence of Lewy bodies in patient brains has not been confirmed, PD with *VPS35* mutation and sporadic PD are thought to share some common pathogenetic mechanisms.

The *VPS35* gene encodes a 796 amino acid protein that acts as a crucial component of the retromer complex, a mediator of the retrograde transport of endosomal proteins to TGN or plasma membranes (102–104). Retromer contains two subprotein complexes: a cargo recognition complex composed of VPS26–VPS29–VPS35 heterotrimer and a membrane-targeting dimer of sorting nexins (SNX1, SNX2, SNX5, SNX6, and SNX32) (105–108). VPS35 is located at the center of the complex and is important for the recognition and binding of the cytoplasmic domain of cargoes for retrograde transport (109). Particularly, retromer is responsible for the retrograde transport of cation-independent mannose 6-phosphate receptor (CI-MPR), a sorting receptor of lysosomal hydrolases including cathepsin D (110). Therefore, the dysfunction of VPS35 or retromer is thought to affect lysosomal activity through impaired delivery of lysosomal hydrolases, and this may also affect α-synuclein clearance, as cathepsin D is one of major enzymes responsible for the degaradation of α-synuclein (41, 43). In relation to PD, it has been reported that PD-associated D620N mutation in VPS35 causes defects in sorting of CI-MPR (111). Also, D620N mutation in VPS35 has been shown to affect retromer binding to the actin-nucleating Wiskott-Aldrich syndrome and SCAR homolog (WASH) complex, an important functional partner of retromer (16, 112).

VPS35 has also been associated with other cellular processes such as autophagy (102). It has been shown that VPS35 regulates macroautophagy by controlling the endosomal localization of WASH complex as well as ATG9a, a multipass transmembrane protein that is considered to regulate the early steps of autophagosome formation (16). Specifically, the transport of ATG9a is affected by D620N mutant VPS35, which then causes the impairment of autophagosome formation. Another study has suggested the role of VPS35 in CMA, where VPS35 mediates endosome-to-Golgi retrieval of LAMP2A receptor (113). Mice with reduced Vps35 level or D620N mutation showed alterations in lysosomal morphology with a decrease in the level of LAMP2A. This may be due to impaired recovery of LAMP2A from the endosome to the Golgi, which then leads to the enhanced degradation at the lysosomes. This reduction in LAMP2A level is expected to cause a decrease in CMA-mediated α-synuclein degradation. Actually, *Vps35*-deficient mice showed multiple PD-like phenotypes such as the accumulation of α-synuclein in DA neurons, reduced level of the catecholamine-synthesizing enzyme tyrosine hydroxylase (TH) and DA transmitters, dystrophic TH-positive neurites/axons, and impaired motor behaviors (113). Another group has reported that lentivirus-mediated overexpression of human wild-type VPS35, but not PD-linked P316S mutant, rescues α-synuclein accumulation as well as α-synuclein-mediated neuronal loss and astrogliosis in α-synuclein transgenic mice (114). In humans, the alterations in the protein levels of CMA markers (LAMP2A and Hsc70) are documented in SNpc and amygdala of PD patients (115). These findings collectively suggest the role of VPS35 as an indirect controller of lysosomes through the regulation of intracellular trafficking of lysosomal enzyme adaptors or multiple autophagic regulators.

## ATP13A2: A Unique Cation Transporter on Lysosomes

Recessive mutations in *ATP13A2* (polyamine-transporting ATPase 13A2), a gene located in a PD-associated locus *PARK9*, have been identified as the genetic cause for Kufor-rakeb syndrome (KRS), which is a type of Parkinsonian syndromes. KRS is clinically characterized by L-DOPA-responsive juvenile parkinsonism as well as cognitive impairment and myoclonus (116), and pathologically characterized by diffuse cerebral and cerebellar atrophy (117). Loss-of-function mutations in *ATP13A2* have additionally been reported to cause neuronal ceroid lipofuscinosis (118, 119). ATP13A2 is a lysosomal P5-type transport ATPase that is involved in the transport of divalent metal cations as well as polyamines on lysosomal membranes (120). Loss of ATP13A2 causes lysosomal accumulation of polyamines (e.g., spermine) and lysosomal rupture, leading to cell toxicity (121). ATP13A2 has also been suggested to regulate multiple cellular functions related to lysosomes, including heavy metal homeostasis and mitochondrial homeostasis (15, 122). For example, a recent study using SH-SY5Y cells, patient-derived fibroblasts and the nematode *C. elegans* has identified a conserved cell protective pathway that counters mitochondrial oxidative stress via ATP13A2-mediated lysosomal spermine export (123).

A number of previous studies have pointed to the essential role of ATP13A2 in the homeostasis of lysosomal function (124). Studies with PD patient-derived mutant ATP13A2 fibroblasts and ATP13A2-knockdown DA neurons have shown that PD-linked mutations in ATP13A2 lead to several lysosomal alterations, including impaired lysosomal acidification, decreased activity of lysosomal enzymes, reduced degradation of lysosomal substrates and defective clearance of autophagosomes (125). Conversely, overexpression of wild-type ATP13A2 in *ATP13A2*-deficient cells restores lysosomal function and prevents cell death (125). Other studies have demonstrated that ATP13A2 regulates endolysosomal cargo sorting through its cytosolic N terminal domain, independent of its catalytic activity (126), and ATP13A2 regulates mTORC1-TFEB pathway together with another PD-associated gene product synaptotagmin 11 (SYT11) to induce autophagy as well as α-synuclein clearance (127). ATP13A2 deficiency and mutation have also been shown to cause the reduction in the level of cathepsin D, a main α-synuclein-degrading enzyme in lysosomes, in human neuroblastoma SH-SY5Y cells and in medaka fish (128).

The relevance of ATP13A2 defects to α-synuclein accumulation has been more directly demonstrated from other studies. Depletion of ATP13A2 in primary cortical neurons using a short hairpin RNA promoted the aggregation of α-synuclein by reducing lysosomal activity, which ultimately led to cell death (15, 129). On the other hand, overexpression of ATP13A2 in α-synuclein-stable SH-SY5Y cells lowered intracellular α-synuclein levels and instead promoted extracellular secretion of α-synuclein (130). Another study has reported that overexpression of ATP13A2 rescued DA neuron degeneration caused by overexpressed α-synuclein in rat primary midbrain cultures and in *C. elegans* (131).

*In vivo, Atp13a2* knockout mice exhibit a neuronal ceroid lipofuscinosis-like phenotype, accumulation of mitochondrial ATP synthase subunit C (132), α-synuclein accumulation, dopaminergic pathology and late-onset sensorimotor deficits (133, 134). More specifically, ATP13A2 deficiency causes dysfunctions in the fusion of autophagic vacuoles with lysosomes as well as the impairment of lysosome-mediated degradation of proteins including α-synuclein (135). Analyses of postmortem PD patient brains have shown the presence of ATP13A2 in the Lewy bodies and a decrease in the levels of lysosomal components including ATP13A2 in DA neurons (125, 136). Although the mutations in *ATP13A2* are rare in humans, these studies have collectively pointed to the important roles of ATP13A2 in ALP that may be involved in the neurodegenerative processes.

## Glucocerebrosidase: The Lysosomal Enzyme Linked to Sporadic PD

Homozygous or compound heterozygous mutations in *GBA1* gene are well-known to cause Gaucher disease (GD), a lysosomal storage disorder, whereas heterozygous mutations that in the homozygous state lead to GD have been reported to increase the risk for developing PD (137–139). Also, a higher incidence of Parkinsonism in patients with GD harboring *GBA1* homozygous mutations has been reported (140, 141). Moreover, a number of genome-wide association studies (GWAS) have identified *GBA1* as a most common genetic risk factor for idiopathic PD (9, 11, 142). Compared to non-*GBA1*-associated PD, *GBA1*-associated PD shows an earlier onset of the disease and a higher prevalence of non-motor symptoms, such as cognitive decline. They also tend to have a family history of dementia, and non-motor symptoms often manifest before the onset of motor symptoms (143, 144). *GBA1* mutations are also a risk factor for dementia with Lewy bodies (DLB) (145, 146), and PD patients with *GBA1* mutation have about a 3-fold higher risk of progressing to dementia than those without mutation (147). They also exhibit a faster progression of visual hallucinations, dysautonomia and motor symptoms, with a resultant decrease in survival rate (143, 145, 148).

*GBA1* gene encodes the lysosomal enzyme glucocerebrosidase (GCase) that hydrolyzes glucosylceramide (GlcCer) to ceramide and glucose. *GBA1* mutations have been shown to cause the reduction in the enzymatic activity of GCase (149, 150) and prevent GCase from reaching the lysosome, causing the accumulation of GlcCer in neurons (151–153). The significant correlation between the severity of the specific *GBA1* mutation and that of clinical phenotypes (e.g., odds ratios for PD, age at onset, risk for dementia) has been reported (145, 147, 154), suggesting major impact of GCase activity in the pathogenetic processes. Importantly, idiopathic PD patients without *GBA1* mutations also showed lower enzymatic activity and levels of GCase in brain tissue samples (155–157) and in dried blood spots (149). The reduction in GCase activity was further demonstrated in PD patient-derived DA neurons without *GBA1* mutations (158, 159). These observations suggest that GCase dysfunction is a common pathogenic mechanism in idiopathic PD.

The reduced function of GCase are expected to contribute to the accumulation of α-synuclein in PD lesions (160). Indeed, treatment with a GCase inhibitor Conduritol B epoxide (CBE) caused a large increase in the levels of α-synuclein, without increasing α-synuclein mRNA, in human neuroblastoma SH-SY5Y cells and in mice (161). The association between reduced GCase and increased α-synuclein is further implicated in human PD postmortem brains (157). The accumulation of GlcCer in neurons as a result of GCase deficiency is thought to promote the formation of toxic α-synuclein aggregates (162), as lipids like GlcCer may strongly interact with α-synuclein and accelerate its fibril formation (163, 164). Another study has suggested a model where α-synuclein deposition and reduced GCase activity may influence each other and form a positive feedback loop that leads to a vicious cycle of disease progression (156).

On the other hand, the activity and function of GCase in microglia or related phagocytic cells have also been focused, as GCase is highly expressed in monocyte lineage cells. In mice, genetic depletion or pharmacological inhibition of GCase caused microglial activation (165, 166). Lower GCase activity was detected in monocytes, but not lymphocytes, from PD patients, when compared with those from healthy subjects (167). Importantly, such reduction in GCase activity was detected in those from patients without *GBA1* mutations. As monocyte lineage cells contain a large number of well-developed lysosomes, it is possible to assume that the dysfunction of lysosomal GCase in these cells greatly influences α-synuclein metabolism.

Recently, much attention has been paid to the relationship between *GBA1* and *LRRK2*. An increasing number of patients harboring both *GBA1* and *LRRK2* mutations have been reported, and these patients tend to develop PD at an earlier age than carriers of *LRRK2* or *GBA1* mutation alone (168–170). These reports suggest the cooperative effect of *GBA1* and *LRRK2* mutations for the development of PD. In experiments using DA neurons derived from PD patients, reduced GCase activity was observed in cells with LRRK2 mutations, and the inhibition of LRRK2 kinase activity restored GCase activity (171). Furthermore, treatment of *GBA1* mutant knock-in astrocytes with LRRK2 kinase inhibitor rescued the lysosomal abnormalities such as pH increase and the reduction in cathepsin B activity (172). These observations collectively suggest that the functions of LRRK2 and GCase in terms of lysosomal regulation are closely interrelated.

## Perspectives on the Therapeutic Strategies Targeting Lysosomes

**Figure 2:**
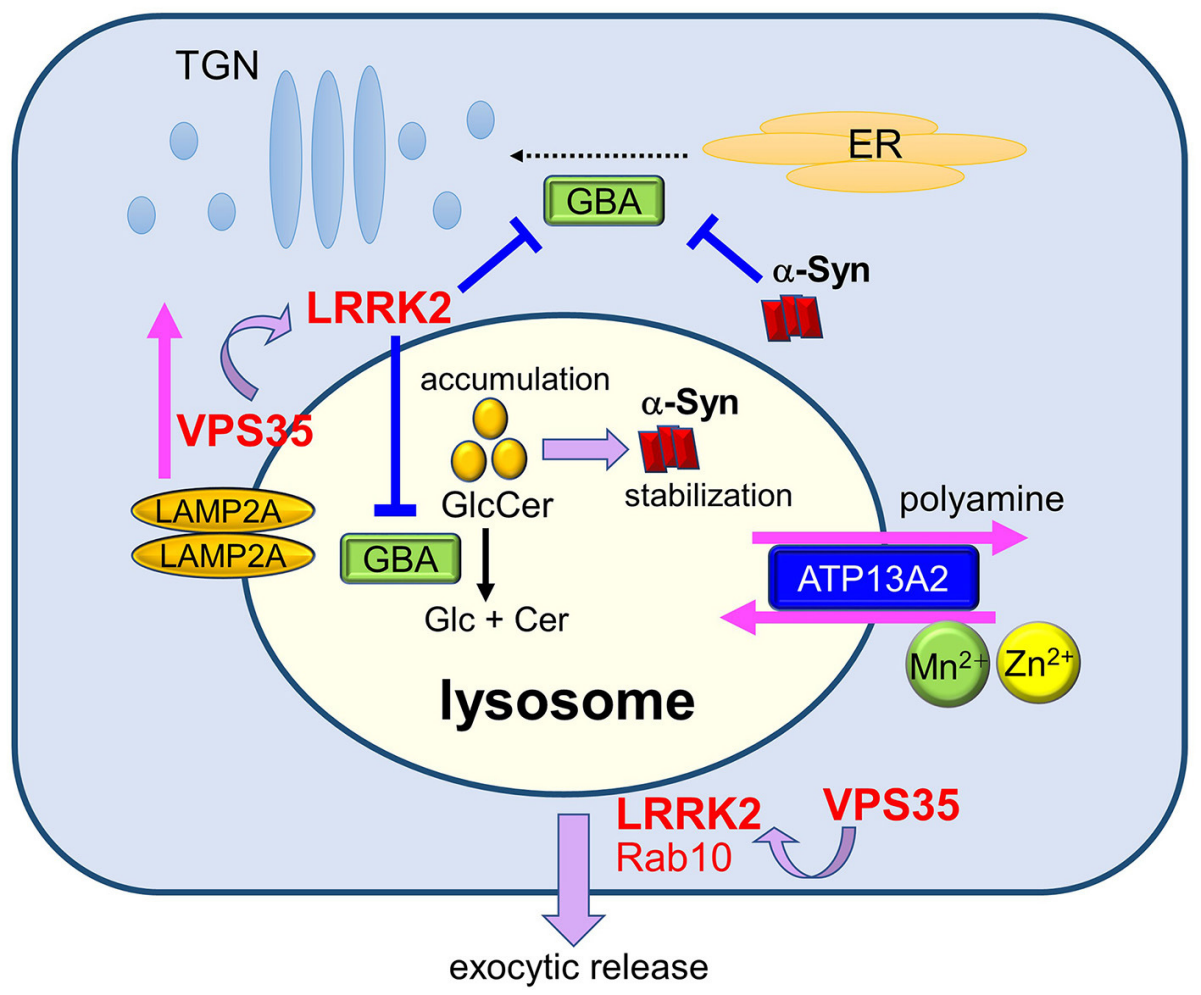
Maintenance of lysosomes by PD gene products and its relevance to PD. Lysosomal homeostasis is regulated by several PD genes such as ATP13A2, LRRK2, GBA, and VPS35. Deficiency in ATP13A2, a P5-type ATPase localized to the lysosomal membranes, is expected to affect lysosomal functions via dysregulated transport of several divalent metal cations and polyamines. LRRK2 functions in the maintenance of stressed lysosomes by facilitating the exocytic release of lysosomal contents together with its substrate Rab10 under lysosomal overload stress. LRRK2 may also negatively regulate the activity of lysosomal hydrolase glucocerebrosidase (GCase). Decreased activity of GCase causes the accumulation of its substrate glucosylceramide (GlcCer), which stabilizes toxic α-synuclein species. Accumulation of α-synuclein has been shown to block ER-to-Golgi trafficking of GCase, causing a further decrease in lysosomal GCase. The retromer component VPS35 mediates the retrieval of a CMA receptor LAMP2A on endolysosomal membranes, and mutation in VPS35 leads to the enhanced degradation of LAMP2A at the lysosomes, causing CMA defects and α-synuclein accumulation. VPS35 mutation also causes the enhancement of LRRK2 kinase activity, which may then affect the lysosomal maintenance and GCase activity.

As described above, ALP can be regulated by PD-associated genes *LRRK2, VPS35, ATP13A2*, and *GBA1* not only individually but also cooperatively. Especially, cooperative maintenance of lysosomes by these genes is considered as one of key mechanisms related to PD (Figure 2). For example, lysosomal morphology under lysosomal overload stress is maintained by LRRK2 kinase activity (82) that is enhanced in cells harboring *VPS35* pathogenic mutation, although the mechanism of enhancement is unclear (97). As lysosomes apparently play important roles in the accumulation and toxicity of α-synuclein, a number of studies have focused on enhancing ALP as a possible therapeutic strategy for α-synucleinopathies (173).

Enhancement of lysosomal activity is one of plausible approaches to facilitate α-synuclein degradation. Among the PD-associated gene products introduced above, GCase has been the most well-studied as a target that contributes to lysosomal activation and α-synuclein metabolism. It has been shown that lysosomal GCase activity can be enhanced by treatment with ambroxol hydrochloride, a clinically used expectorant drug and an effective pharmacological chaperone for GCase (174–176). Oral administration of ambroxol to wild-type and α-synuclein transgenic mice caused the increase in brain GCase activity as well as the reduction in the levels of total and phosphorylated α-synuclein (177). Amboxol administration in rats also resulted in the restoration of decreased GCase activity and the decrease of α-synuclein pathology that were induced by 6-hydroxydopamine (6-OHDA) treatment (178). Additionally, oral administration of another molecular chaperone for GCase, AT2101 (afegostat-tartrate, isofagomine), to α-synuclein transgenic mice improved motor and non-motor function, abolished microglial response in the substantia nigra, and reduced the number of small α-synuclein aggregates (179). Adeno-associated virus (AAV)-mediated overexpression of GCase in hippocampus ameliorated α-synuclein accumulation as well as cognitive impairment in transgenic mice expressing mutant GCase (D409V/D409V) or A53T α-synucein (180, 181). Using the same mice models, the researchers have also shown that the administration of a brain-penetrant inhibitor of GlcCer synthase (GCS), GZ667161, ameliorated α-synuclein accumulation and cognitive deficits (182). These reports indicated that proper GlcCer metabolism is important to control α-synuclein accumulation.

Farnesyltransferease inhibitors (FTIs) are recently attracting significant attention as a promising lysosomal activator. It has been reported that FTI treatment in α-synuclein transgenic mice enhanced GCase activity and rescued pathological α-synuclein aggregation (49). FTI treatment has also been reported to reduce tau pathology in tauopathy model mice by activating lysosomes (183). Importantly, one of FTIs, lonafarnib, has been approved by FDA very recently for the treatment of Hutchinson-Gilford progeria syndrome, a rare and fatal premature aging disease (184). Thus, it will be of particular interest to see if such therapeutic strategies are actually effective in the treatment of PD or related neurodegenerative disorders.

Another plausible approach to activate lysosomes is the expression of transcription factor EB (TFEB), a master transcriptional regulator of ALP. Overexpression of TFEB has been shown to rescue midbrain DA neurons from α-synuclein-induced toxicity in transgenic rat models (185). In addition to α-synuclein, overexpression of constitutively active TFEB has been shown to reduce protein aggregates in old quiescent neural stem cells (qNSCs) (186) and in p53-induced senescent fibroblast cells (187). Nuclear translocation of TFEB is induced by inhibition of mammalian target of rapamycin (mTOR) (188), a well-known negative regulator of macroautophagy and ALP, and therefore mTOR inhibition has also been focused as a promising strategy. Intra-cerebral infusion of an mTOR inhibitor rapamycin for 2 weeks in α-synuclein transgenic mice resulted in reduced accumulation of α-synuclein (189), and long-term feeding a rapamycin diet (~24 weeks) improved motor performance in A53T α-synuclein transgenic mice (190). However, due to the side effects of rapamycin that have been noted to be problematic with long-term use (191), the use of rapamycin in the treatment of PD is expected to be challenging.

On the other hand, an mTOR-independent activator of autophagy, trehalose, has been shown to activate macroautophagy and enhance the clearance of wild-type or mutant forms of α-synuclein (192–195). Mechanistically, trehalose has been shown to activate macroautophagy by inhibiting the glucose transporter SLC2A, which ultimately leads to the activation of an energy-sensing kinase AMPK that stimulates autophagy (196). Oral administration of trehalose to A53T α-synuclein transgenic mice for 1 week induced macroautophagy and reduced the level of insoluble α-synuclein in the brain (197). Similarly, oral administration of trahalose to AAV-based rat model expressing A53T α-synuclein for 6 weeks caused a significant attenuation in α-synuclein-mediated motor deficits and DA neurodegeneration as well as α-synuclein accumulation (198). In addition to trehalose, a tyrosine kinase inhibitor nilotinib is another drug that stimulates autophagy by activating AMPK (199); chronic administration of nilotinib for 3–6 weeks in human A53T α-synuclein transgenic mice resulted in the decrease of α-synuclein levels, suppression of DA neuronal loss and improvement of motor behavior (200).

Activation of the CMA pathway is considered as an alternative strategy to increase the clearance of α-synuclein. Overexpression of LAMP2A has been shown to upregulate CMA, decrease α-synuclein accumulation and protect against α-synuclein toxicity in human neuroblastoma SH-SY5Y cells, rat primary cortical neurons, and nigral dopaminergic neurons *in vivo* (201). Inhibition of signaling through retinoic acid receptor α (RARα), a negative regulator of CMA, has also been focused; treatment with the RARα inhibitor all-*trans*-retinoic acid and its synthetic derivatives has been shown to activate CMA and protect against oxidative stress and proteotoxicity in cells (202). A specific subset of miRNAs that downregulate CMA has also been identified (203), and treatment with Geniposide, a bioactive iridoid glycoside that acts as a down-regulator of miRNAs especially for miR-21, increased LAMP2A expression and reduced α-synuclein levels in SH-SY5Y cells and MPTP-treated PD model mice (204).

In conclusion, a variety of strategies that aim to activate ALP have been developed and shown to modulate α-synuclein accumulation as well as PD-related phenotypes. The strategies that were tested for *in vivo* phenotypic modulation are summarized in Table 1. Several of the compounds used in these strategies are now being examined in clinical trials for PD and related disorders [e.g., ambroxol (205) and nilotinib (206, 207), *see* *ClinicalTrials.gov*]. These compounds or related products with similar mechanisms is expected to be available in the future as disease-modifying therapies. Moreover, as overviewed above, ALP is regulated in various ways by PD gene products—including LRRK2, VPS35, ATP13A2, GCase, and other risk factors not mentioned in this review—and among these, not only GCase (activator, ambroxol) but also LRRK2 (inhibitor) are being targeted in clinical trials (208). Further clarification of the functional relationships among PD-causing genes and their regulation to ALP may lead to the proposal of new therapeutic targets. It is hoped that further basic analysis of cellular and animal models, such as those described in this review, will accelerate the development of fundamental therapeutic agents.

**Table 1:** Strategies to enhance lysosomal activity for the modulation of PD-related phenotypes *in vivo*.

**Target**	**Strategy**	**Effects**	**Reference**
GCase	Oral administration of GCase chaperones (ambroxol, AT2101)	Reduction of total- and phospho-α-synuclein Decrease of 6-OHDA-induced α-syn pathology Reduction of small α-syn aggregates (AT2101)	(177) (178) (179)
GCase	Overexpression of GCase	Amelioration of α-syn accumulation and cognitive impairment in Gba1-D409V or α-syn-A53T Tg mice	(180) (181)
GlcCer synthase	GlcCer synthase inhibitor (GZ667161) administration	Amelioration of α-syn accumulation and cognitive impairment in Gba1-D409V or α-syn-A53T Tg mice	(182)
Farnesyltransferase	Farnesyltransferase inhibitor (FTI) treatment	sReduction of pathological α-synuclein in Tg mice Increase of GCase activity Reduction of tau pathology	(49) (183)
TFEB	Overexpression of TFEB	Protection of DA neurons from α-syn toxicity in Tg rats	(185)
mTOR	Rapamycin treatment	Reduction of α-synuclein accumulation (2 weeks) Improvement of motor function (24 weeks)	(189) (190)
Autophagy-AMPK	Trehalose treatment	Reduction of insoluble α-synuclein (1 week) Attenuation of motor deficits, degeneration and α-syn deposition (6-weeks)	(197) (198)
Autophagy-AMPK	Nilotinib treatment	Reduction of α-syn levels, suppression of DA neuron loss and motor deficits in α-syn-A53T Tg mice	(200)
CMA	Overexpression of LAMP2A	Complete restoration of α-syn-mediated nigrostriatal degeneration in AAV-α-syn rats	(201)
CMA	Geniposide treatment	Decrease of α-syn levels and increase of LAMP2A in MPTP-treated mice	(204)

## Author Contributions

TA and TK conceived and wrote the article. All authors contributed to the article and approved the submitted version.

## Conflict of Interest

The authors declare that the research was conducted in the absence of any commercial or financial relationships that could be construed as a potential conflict of interest.
